# Organellar Introns in Fungi, Algae, and Plants

**DOI:** 10.3390/cells10082001

**Published:** 2021-08-06

**Authors:** Jigeesha Mukhopadhyay, Georg Hausner

**Affiliations:** Department of Microbiology, University of Manitoba, Winnipeg, MB R3T 2N2, Canada; mukhopaj@myumanitoba.ca

**Keywords:** organellar intron, Group I intron, Group II intron, splicing, homing, retrohoming, twintron

## Abstract

Introns are ubiquitous in eukaryotic genomes and have long been considered as ‘junk RNA’ but the huge energy expenditure in their transcription, removal, and degradation indicate that they may have functional significance and can offer evolutionary advantages. In fungi, plants and algae introns make a significant contribution to the size of the organellar genomes. Organellar introns are classified as catalytic self-splicing introns that can be categorized as either Group I or Group II introns. There are some biases, with Group I introns being more frequently encountered in fungal mitochondrial genomes, whereas among plants Group II introns dominate within the mitochondrial and chloroplast genomes. Organellar introns can encode a variety of proteins, such as maturases, homing endonucleases, reverse transcriptases, and, in some cases, ribosomal proteins, along with other novel open reading frames. Although organellar introns are viewed to be ribozymes, they do interact with various intron- or nuclear genome-encoded protein factors that assist in the intron RNA to fold into competent splicing structures, or facilitate the turn-over of intron RNAs to prevent reverse splicing. Organellar introns are also known to be involved in non-canonical splicing, such as backsplicing and trans-splicing which can result in novel splicing products or, in some instances, compensate for the fragmentation of genes by recombination events. In organellar genomes, Group I and II introns may exist in nested intronic arrangements, such as introns within introns, referred to as twintrons, where splicing of the external intron may be dependent on splicing of the internal intron. These nested or complex introns, with two or three-component intron modules, are being explored as platforms for alternative splicing and their possible function as molecular switches for modulating gene expression which could be potentially applied towards heterologous gene expression. This review explores recent findings on organellar Group I and II introns, focusing on splicing and mobility mechanisms aided by associated intron/nuclear encoded proteins and their potential roles in organellar gene expression and cross talk between nuclear and organellar genomes. Potential application for these types of elements in biotechnology are also discussed.

## 1. Introduction

Introns, or intervening sequences, are segments that are transcribed but removed from a transcript before translation can proceed. There are several categories of introns, such as tRNA introns, nuclear spliceosomal introns, and Group I and Group II introns [1]. Introns are ubiquitous in eukaryotic genomes and have long been considered as ‘junk RNA’ but the huge energy expenditure in their transcription, removal, and degradation indicate that they may offer a significant evolutionary advantage [2,3]. It has been speculated that Group I and Group II introns which are potential ribozymes, could have evolved in the RNA world [4] whereas spliceosomal introns are potentially derived from Group II introns that colonized the nuclear genome during eukaryogenesis [5,6,7]. In comparison to the impact of nuclear spliceosomal introns on gene regulation and expression, less is known about the contribution of organellar Group I and II introns towards cellular processes.

Organellar genomes can be quite variable in size due to the absence and presence of introns, however, little is known about the mechanisms that promote intron gain and loss and why in some instances, the organellar genomes are devoid of introns, or in extreme examples where introns comprise most of the nucleotides that makeup the genome [8,9,10]. Organellar introns are potential mobile elements that can self-splice and, thus, minimize their impact on the host genes they have invaded. However, these introns can encode protein factors that catalyze their mobility and promote splicing. Additional genome encoded factors also appear to have been co-opted to enhance splicing and mobility. Although models have been developed that suggest that organellar introns are neutral elements, their reliance on genome encoded factors or in some instances, trans-acting intron encoded splicing factors would suggest that more complex evolutionary models may be needed to explain the biology of these elements [11,12]. The splicing of organellar introns could be a rate limiting step that can affect the expression of the genes in which they are embedded [6]. Complex organellar introns composed of two or three intron modules have been characterized in some genomes and these might be platforms for alternative splicing (AS) or serve as molecular switches for modulating gene expression [13,14,15]. Cis- and trans-splicing combined with the possibility of these elements to transpose to new locations within genomes or between genomes provides a mechanism for generating genetic diversity. Group I and Group II intron are “building blocks” and these mobile or self-splicing modules can lead to the formation of complex-introns, variable gene architectures, and promote organellar genome evolution [16].

## 2. Organellar Introns: Group I and Group II Introns

Organellar introns are mainly classified as self-splicing introns that are either Group I and II type introns, based on differences in their splicing pathways and RNA secondary structural features. Sequence conservation among Group I or Group II introns is very low, but conservation among these elements can be found at the structural level [11,17,18,19]. Group I and II introns can have embedded open reading frames (ORFs) that encode for intron encoded proteins (IEPs) [20,21,22,23,24,25].

**Figure 1:**
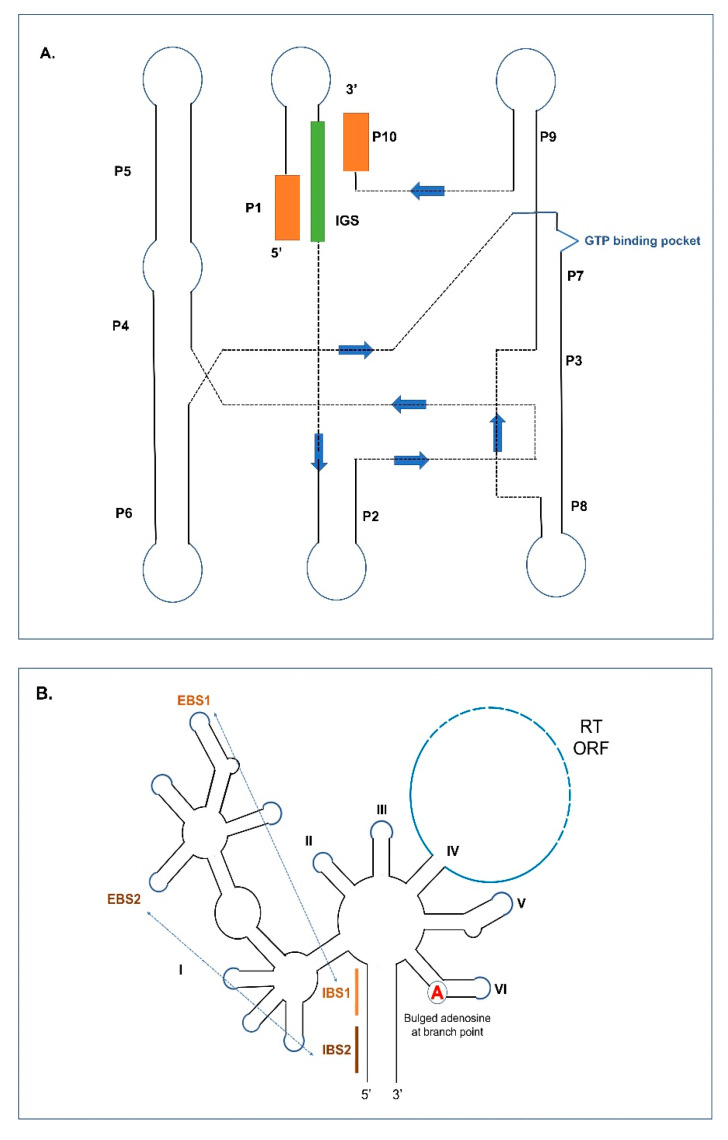
Secondary structure models for Group I and II introns. (**A**) Generic secondary structure representation of Group I intron showing the stem regions (black), loop regions (blue), 5′ and 3′ exon sequences (orange), and the internal guide sequence or IGS (green). The ten pairing regions (P1–P10) are indicated of which, the P1–P10 interaction is usually critical in determining the splice site. The guanosine-5′-triphosphate (GTP) binding pocket within the P7 helix is also indicated. (**B**) Generic secondary structure of Group II intron showing the six interacting domains (DI-DVI) with the RT-ORF protruding out of the domain IV (blue). Intron and exon binding sequences and the bulged adenosine (A, red) at the branch point are utilized in Group II intron splicing; interactions are indicated as IBS1-EBS1 (orange) and IBS2-EBS2 (brown).

Group I and II introns at the RNA level assume RNA folds that generate the active sites needed for the splicing reactions to be initiated (Figure 1A,B). Group I intron RNAs contain helical or paired regions referred to as P1 to P10 (some Group I introns can have additional paired regions) with loop segments connecting the helical regions. The P1 helix includes a segment referred to as the internal guide sequence (IGS) which promotes the formation of the P10 helix, which ultimately coordinates the positions of the upstream and downstream exon sequences. The P7 helix includes a GTP binding pocket that recruits the GTP needed to initiate the splicing process. Group I intron RNAs can self-splice by forming RNA folds where the P3 and P7 plus proximal P4, P5, P6, and P9 paired helical domains comprise the catalytic core components and the P1 and P10 helices generate the substrate domain. This RNA configuration brings the splice sites (intron/exon junctions) into proximity with each other. Group I introns are classified into various subtypes based on variations within the primary, secondary, and tertiary structures [9,26,27,28,29,30]. These subtypes (IA to IE) can be further subdivided (e.g., IA1, IC3, etc.) [31,32,33].

Group II intron RNA can be visualized as 6 stem-loop domains (referred to as domains DI to DVI) emerging from a central wheel. When ORFs are present they tend to be embedded within DIV. Domain I is the largest domain and is referred to as the scaffold domain that in part co-ordinates the folding and interactions of the other components of the Group II intron RNA. So called exon binding sequences (EBSs) are embedded within DI that can interact via H-bonding with intron binding sequences (IBSs) that are located within the exons flanking the Group II intron. The bulged adenine (A), referred to as the branch point, is located in DVI. DV is the most conserved segment of Group II introns, and it is important in facilitating the interactions of DVI with the upstream intron–exon junction by coordinating the position of two Mg^2+^ ions that permits the 2′OH Group on the budged adenine to destabilize the phosphodiester bond (PDE) that links the upstream exon with the intron [9,34,35,36,37,38,39]. Like Group I introns, sequence conservation among Group II introns is minimal except for DV and the intron boundaries, with GUGYG and AY (Y = pyrimidines) defining the 5′ and 3′ ends, respectively. Various subtypes of Group II intron have been recognized based on structural features and the nature of EBS and IBS interactions [40,41]. Group II intron RNAs found in organellar genomes can be classified into two major subgroups: IIA and IIB. These can be further divided into IIA1, IIA2, IIB1, and IIB2 [42,43,44,45]. Most mitochondrial Group II introns can be assigned to subgroup IIA1, and many chloroplast Group II introns can be assigned to subgroup IIB [46]. Group II-like introns, which historically were classified as Group III introns, lack the standard Group II core structure, either due to loss of domains DI-IV or massive divergence from related Group II introns [47,48]. Such degenerate Group II introns were identified in mRNA genes of chloroplasts in euglenoid protists, having a conventional Group II-type DVI domain with a bulged A residue, a streamlined version of DI, and absence of DII-DV [14].

## 3. Distribution and Impact of Introns on Organellar Genome Architecture

Group I introns are widespread and have been reported from nuclear rDNA (18S and 26S RNA genes) in fungi, protozoans, metazoans [36,37,38,39,40,49,50,51,52,53,54,55,56,57,58], bacterial genomes, phages and viruses, archaeal genomes, and they are encountered frequently in mitochondrial and plastid genomes (reviewed in [59]). With regard to metazoans, Group I introns have been noted in the mitochondrial genomes in members of Placozoa, Anthozoa, and Porifera [54,56,57]. Noteworthy recent discoveries include putative Group I introns in the nuclear rDNA internal transcribed spacers (ITS) in several fungi and closely related unicellular organisms, such as *Mitosporidium* [60], *Polychytrium* [61], *Amoeboaphelidium,* and *Nuclearia* [61]. These ITS Group I introns have been named spintrons (spacer introns) and examples of these introns have also been reported in sharks [62]. 

Group II introns also have a wide phylogenetic distribution and are found in bacteria and the organellar genomes of plants, fungi, protists, and animals [54,63,64]. Though they are completely absent from nuclear eukaryotic genomes, they are hypothesized as the ancestors of spliceosomal introns during eukaryotic evolution ([65]; Figure 1). There are rare occurrences of Group II introns in some sponges [54] and bilaterian animal genomes, an example being a *cox1* gene-associated Group II intron in the mitochondrial genome of the carnivorous polychaete *Nephtys* sp. [66]. In bacterial and archaeal genomes, Group II introns have been identified which either entirely lack an ORF or encode novel RT ORFs and this indicates that certain lineages have adapted unusual RNA features, beyond being a retroelement [67]. The vast majority of mitochondrial introns in angiosperms are of the Group II type [68]. 

**Figure 2:**
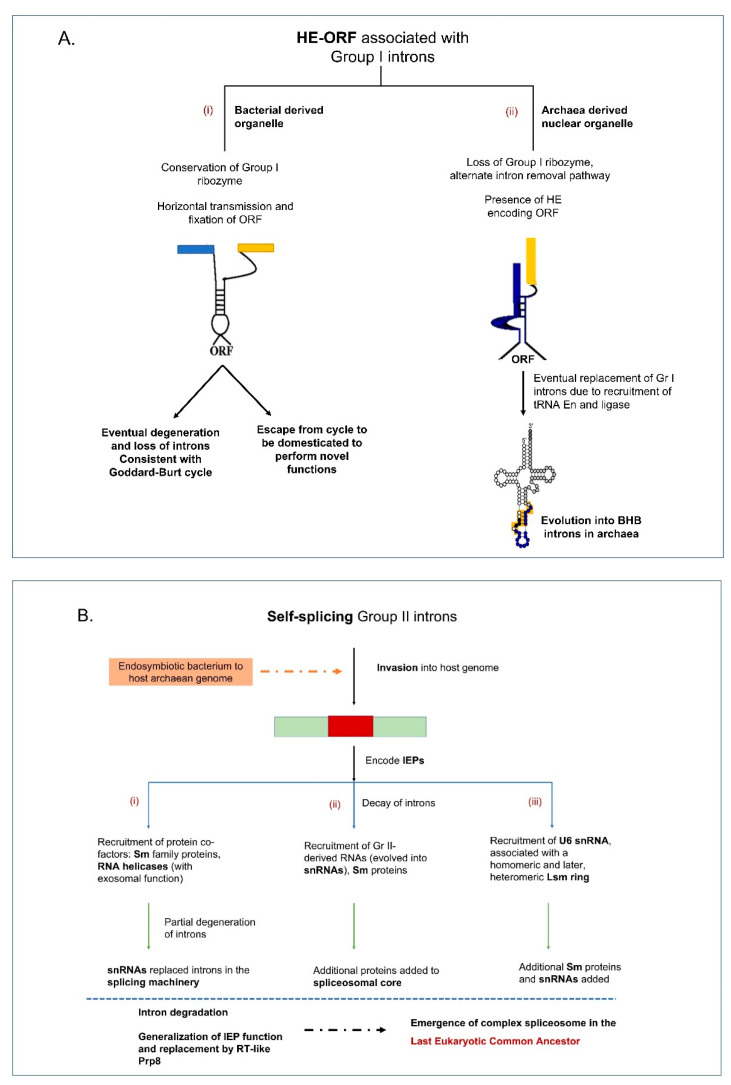
Probable evolutionary fates of self-splicing introns. (**A**) Homing endonuclease-open reading frame (HE-ORF) of Group I introns follow the Goddard–Burt life cycle, with slightly altered fates in bacterial derived organelles (mitochondria and chloroplasts) and archaeal derived nuclear genomes. (i) For bacterial derived organelles, the Group I ribozyme is conserved in the absence of tRNA endonuclease and ligase and is eventually lost during evolution when the intron function is lost or becomes redundant; (ii) For archaea derived nuclear genomes, the Group I ribozyme ability was lost in favor of an alternate intron removal pathway recruiting the tRNA endonuclease and ligase. Because of this recruitment, the Group I introns in archaea have probably evolved into BHB introns, both can encode homing endonucleases and these intron types can be distinguished by computational approaches [71]. (**B**) Origin of spliceosome from Group II introns involves domestication of the self-splicing introns (during eukaryogenesis) and recruitment of protein components into the spliceosome. The order of intron decay and protein recruitment is debatable; (i) Recruitment-first theory: recruitment of Smith antigen (Sm) protein cofactors, followed by RNA helicase (with probable exosomal function); subsequent partial degeneration of the introns [74]; (ii) Decay-first theory: decay of self-splicing introns, requiring the recruitment of Group II-derived RNAs, which evolved into the small nuclear RNAs (snRNAs), and associated Sm proteins [5]; (iii) ‘Frozen event’ theory: starts with RNA components, at least U6 snRNA, associated with a homomeric, and later heteromeric Sm-like (Lsm) ring whose interaction can be seen as a ‘frozen event’; addition of other snRNAs, accompanied by the Sm ring. Irrespective of the order of events, eventually intron degradation and substitution of Group II intron activity by reverse transcriptase (RT)-like pre-mRNA splicing factor 8 (Prp8) activity led to the emergence of the complex spliceosome in the last common eukaryotic ancestor (LECA) [75].

It is speculated that Group I introns are potential ancestors to the tRNA (Bulge–helix–bulge) introns and Group II introns are potential ancestors to the nuclear spliceosomal introns [69] (Figure 2A,B). Bulge–helix–bulge (BHB) introns are found within the tRNA genes (tDNAs) of the archaea and they may have evolved from Group I intron ancestors but recruited tRNA endonucleases and ligases for intron removal [70]. The canonical BHB structural motif is usually found at the exon-intron boundaries within the anticodon loop of pre-tRNA consisting of a 4 base-pair (bp) helix flanked on each 3′ side with 3 nucleotides in the bulge [71]. There have been reports of possible tRNA introns in fungal mitochondrial genomes but they do not appear to fit into any known category of intron-type elements [72]. Within chloroplast genomes, Group II introns are frequently embedded within tRNA genes [73].

Among the fungi, Group I introns are more frequently encountered compared to Group II introns, however Group I introns are not widespread among the Chlorophyta organellar genomes. Lichenization and maintenance of lichen symbiosis might have facilitated horizontal transfer of introns between symbionts, an example being chloroplast Group I introns in the chlorophyte algae, Trebouxiophyceae [76]. There is some speculation that plant organellar Group I introns could have been horizontally transferred from fungal organellar genomes (see Section 8 for more details). It has also been speculated that introns located in the metazoan mitochondrial genomes may have been acquired via horizontal gene transfer (HGT) possibly mediated by viruses or by predation on fungi by some animals [57,59].

Group II introns are more commonly distributed in both the mitochondria and chloroplast of the Chlorophyta. Organellar lineages of Group II introns have been recognized that are the mitochondria-like lineage (ML) and the chloroplast-like lineage (CL) and this phylogenetic distribution is viewed to be indicative of their endosymbiotic origins of the chloroplasts and mitochondria and the co-evolution of their intron-encoded proteins with the ribozyme intron RNA partners [77,78]. In plants, mitochondrial and chloroplast gene expression involves splicing of introns from the coding regions. In both chloroplast and mitochondrial genomes of the angiosperms, Group II introns tend to dominate, and these are usually found associated with the cytochrome (cyt) complexes and within ribosomal RNA and tRNA genes. 

There have been reports demonstrating that organellar introns can promote genome size variation and genome rearrangement [79,80]. For example, among fungal mitogenomes characterized for *Tramates* species (*Polyporales*, Basidiomycota), a huge variation was seen in intron numbers between different species due to intron gain and loss events, along with the discovery of novel introns, not detected before in Basidiomycota [81]. A unique gene arrangement was also reported in the mitogenome of *Turbinellus floccosus* (Basidiomycota), with a specific bias for intron insertion downstream of a T base, preferably in the downstream site of a GT or AT, which might be a result of insertion or homing biased for specific conserved sites [82]. 

Plastid genomes have been studied in chlorophyte algae such as *Chlamydomonas reinhardtii* and *Euglena gracilis* (green lineage), with the latter having a pervasive invasion of 150 introns. In contrast, in *C. reinhardtii,* hundreds of small, dispersed repeats and diverging gene fragments contribute to the genome architecture [83]. In non-green alga (red lineage), non-coding sequences and introns are rare and gene density is overall uniform and they have retained chloroplast DNAs only to utilize plastids for additional functions and expanded coding capacity which might be advantageous in specific environments instead of transferring the chloroplast genes back to the nucleus [84]. A study on the *photosystem II protein DI (psbA*) gene of *Euglena myxocylindracea* gave evidence for the horizontal transfer of a self-splicing, mobile Group II intron from a cyanobacteria into its chloroplast genome [85]. Comparative studies were conducted between *Mesostigma viride* (green algae belonging to Mesostigmatales) and land plant chloroplast genome sequences [86]. Complete chloroplast DNA sequencing of two members of the Zygnematales that belong to distinct lineages, *Staurastrum punctulatum* and *Zygnema circumcarinatum,* showed that high variation in gene order and intron content arises out of rRNA-encoding inverted repeat (IR) loss event. Similarly, in streptophyte algae, IR loss and expansion, and extensive gene shuffling in Klebsormidiophyceae and Zygnematophyceae led to huge variations in the chloroplast genome, resulting in events of Group II intron loss and gain of putative foreign genes, especially of viral or phage origin [87].

In Chlorophyceae, extensive rearrangement of chloroplast genes also takes place along with IR loss or gain through lateral transfer of mobile elements from unknown donors [88]. Comparative analysis between the chloroplast genome of *Oedocladium carolinianum* with the IR-containing genome of *Oedogonium cardiacum* showed that they differ extensively in intron content, abundance of dispersed repeats, and putative coding sequences, with high proliferation of Group II introns in *Oedocladium* making it 7.9 kbp bigger than *Oedogonium*.

Mitochondrial genome evolution in cryptophytes is also of great interest, and a recent report on seven genomes of this genera helped unraveling the broad evolution of organelle genomes [89]. The *cob* and *cox1* gene sequences suggested that introns have been recently acquired during cryptophyte evolution, throughout which extensive gene rearrangements occurred, especially associated with hairpin-containing mobile genetic elements, tRNAs with palindromic sequences, and tandem repeat sequences.

Unlike IR structures which are prevalent in most plant plastomes, early vascular plants like *Selaginella*, have direct repeats (DR). Comparative analysis of lycophytes provided an overview of phylogenetic relationships between *Selaginella* and related lycophytes, and dynamic rearrangements between IR and DR yielded unique genomic features in its plastome [90]. A genus-wide GC bias, high occurrence of RNA editing, a near complete loss of IR, presence of DR blocks are some of the salient features of the *Selaginella* plastome, which was also significantly compacted to presumably provide adaptive advantages in dry environments through the reduction in Group II intron content and the loss or pseudogenization of *NADH* dehydrogenase *(ndh*) genes. Evolutionary comparative analyses on 115 plastomes from green algae, bryophytes, pteridophytes (spore bearing vascular plants), gymnosperms, and angiosperms provided a comprehensive overview of intron length variation and intron phase polymorphism among plants [91]. This study showed that introns were present in 18 protein coding genes, six tRNA genes, and one rrn gene, with a bias of introns located in phase 0 positions (i.e., introns do not disrupt codons) in protein coding genes. The comparative study also showed that the trnK-UUU intron (a group II intron that encodes matK) was present in most algae and seed plants but this intron appears to have been lost in chlorophyte algae and monilophyte ferns. However, in the latter two groups, matK was retained as a free-standing gene [91]. 

Phylogenetic analyses based on mitochondrial DNA provide insights into sister-group relationship between green algae of the order Charales and land plants since mitochondrial DNA has undergone many changes during the evolution of land plants and differs from their green algal counterparts in terms of size and presence of diverse mobile elements and spacers. The common ancestor of green algae and land plants harbored a tightly packed, gene-rich, and relatively intron-poor mitochondrial genome. It is speculated that Group II introns have spread into new mitochondrial DNA sites from this ancestral mitogenome during the evolution of bryophytes and charalean green algae, accounting for part of the intron diversity found in Chara and land plant mitochondria [84]. A similar sister-group relationship was also reported between Ulvophyceae and Chlorophyceae (green lineage) with their mitogenomes containing numerous small, dispersed repeats in intergenic regions and introns [92].

Studies on the green tide forming alga *Ulva compressa* showed multiple intraspecific variations among the ulvophycean mitogenomes [93]. These genomes had variable intron and intronic ORF content. The Group I introns in *Ulva* species were most likely inherited vertically from a common ancestor in the Ulvales/Ulotrichales lineage, the Group II introns possibly accumulated from multiple horizontal transfers to specific insertion sites in the *Ulva* lineage. The wide intraspecific variations in ulvophycean mitogenomes resulted from insertion of foreign DNA fragments, frequent gain or loss of both Group I and II introns, enrichment of repeat sequences, and rearrangement by inversion of a syntenic block with eight collinear genes. Sequencing of mitogenomes from a lichen-symbiont microalga, *Trebouxia* sp. TR9 provides another example of genome expansion due to intergenic spacers and enrichment with Group II introns [94]. 

Mitochondrial genomes in siphonous green algae of the order Bryopsidales, *Caulerpa lentillifera,* and *Ostreobium quekettii* were sequences and compared with their chloroplast genomes to investigate their evolution [95]. Genome expansion was observed in the mitogenomes due to Group II intron proliferation, but the chloroplast genomes stayed compact. Thus, evolutionary forces that impact the size and architecture on the two organellar genomes are possibly independent from each other (see [96]), with considerable variation in gene and intron densities and lack of evidence on intron exchange between the two organellar compartments among the studied members of the Bryopsidales. Non-canonical Group II introns were also recently identified through long-read RNA sequencing in *C. lentillifera*, with a deviant secondary structure and intronic ORFs lacking known splicing or mobility domains, however maintaining the ability for precise splicing [97]. This study provided novel insights into structural variations, expression of polycistronic transcripts, freestanding ORFs, and fragmented genes in algal chloroplast genomes, along with uncharacterized Group II categories and IEPs.

## 4. Splicing in Group I and II Introns

**Figure 3:**
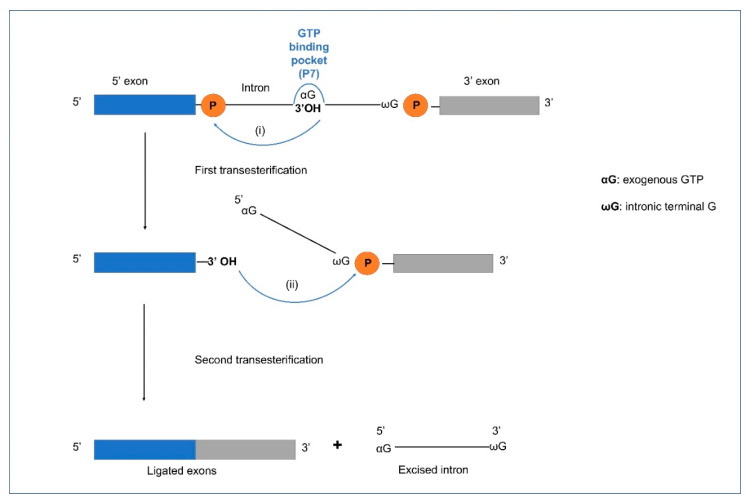
Schematic representations of splicing for Group I introns. The splicing pathway of Group I introns consists of two sequential transesterification pathways. The first reaction is initiated by the 3′ OH group of an exogenous GTP (αG) that docks into the GTP-binding pocket of the P7 helix and the 3′ OH group attacks the 5′ splice site (SS). In the second reaction, the 3′ OH of the released 5′ exon attacks the phosphodiester (PDE) bond between the intronic terminal G (ωG) and the 3′ exon, resulting in the liberation of the introns and the ligation of the exons.

The basic splicing reaction of Group I introns (Figure 3) is transesterification involving two nucleophilic attacks, first by a free guanosine (G) nucleoside co-factor, releasing the 3′OH on the upstream exon and the second attack (mediated by the 3′OH on the upstream exon), excising the intron, and joining the flanking exon sequences. The exons are ligated to form the spliced mature RNA. Group I introns may also transpose to new sites (ectopic integration) involving RNA intermediates through the process of reverse splicing. This involves a released Group I intron RNA that can insert into a homologous or heterologous RNA facilitated by complementary base pairing between the intron IGS and complementary exon RNA sequences. Birgisdottir and Johansen (2005) investigated the site-specificity of reverse splicing of a Group I intron located in ribosomal RNA. They used the twin-ribozyme intron (with two active sites derived from two Group I intron modules) from the myxomycete *Didymium iridis* to show that Group I introns can target rRNA molecules in a site-specific manner through reverse splicing [98]. This study showed that reverse splicing is a mechanism that provides Group I introns with a mobility mechanism.

**Figure 4:**
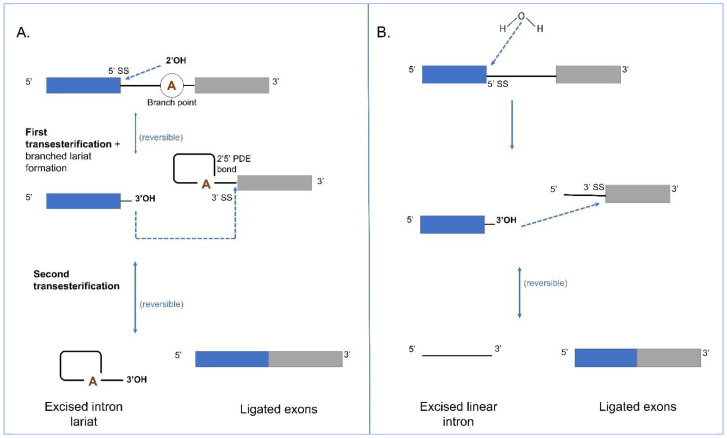
Schematic representation of splicing for Group II introns. (**A**) In the “branching” pathway of splicing, there are two sequential transesterification reactions. In the first reversible reaction, the 2′ OH of an internal branch point (the bulged A), located near the 3′ end of the intron attacks the 5′ splice site (SS), producing a branched lariat intermediate, stabilized by a 2′5′ linkage. In the second reversible reaction, the newly released 3′ OH of the 5′ exon attacks the 3′ SS, resulting in the ligation of the 5′ and 3′ exons and the excision of the intron lariat. (**B**) In the “hydrolytic” pathway of splicing, a water molecule or hydroxyl residue initiates the first attack on the 5′ SS, releasing a linear RNA molecule. In the second reversible reaction, the newly released 3′ OH of the 5′ exon attacks the 3′ SS similar to the branching pathway, resulting in the ligation of the 5′ and 3′ exons and the excision of the linear intron RNA molecule.

The Group II intron splicing pathway (Figure 4A) also consists of two trans-esterification reactions. In the first reaction, the 2′OH of an internal branch-point A, located near the 3′ end of the intron attacks the 5′ splice site (ss). This reaction, referred to as the “branching pathway”, releases the 5′ exon and produces a branched lariat intermediate that is stabilized by a 5′2′ linkage at the branch-point A. In the second reaction, the newly released 3′OH of the 5′ exon attacks the 3′ ss, resulting in the ligation of the 5′ and 3′ exons and excision of the intron lariat. The reversibility of the transesterification reactions allows the potential of reverse splicing of the excised intron into RNA or DNA molecules containing sequences that could base pair with the intron and exon binding sequences [44,99]. For splicing to occur, the 5′ and 3′ exon sequences must be recognized by intron sequences in order to position the scissile phosphates in the active site [9]. In the pre-catalytic structure, there is coordinated docking of the branch point and both splice sites in the active site before splicing [100]. This coordination requires two Mg^2+^ ions. Interestingly, in both Group II introns and spliceosomal introns, the removal of the branched helix from the active site is required to allow the 3′ exon to enter and for the second step of splicing to proceed [101]. In the post-catalytic lariat structure, the nucleobase component of the bulged A is disordered, and the lariat-PDE bond is located approximately 20 Å from the active site indicating that, after the first step catalysis, necessary reactants and products of the first step move out of the active site to make room for reactants of the second step. The 5′ and 3′ ends of the intron, in close proximity to the lariat bond, interact with each other through the first nucleotide, forming a non-canonical base pair with nucleotide A [102]. Group II intron splicing is facilitated by intron and host genome encoded factors. Some Group II introns encode a RT-like protein and these contain a maturase domain that can recognize its parent intron RNA by forming strong and highly specific interactions with a specific RNA segment that is located within D4 of the intron. This protein-RNA interaction orients the IEP in a spatial position that facilitates splicing [103]. In some instances, Group II introns can be spliced out by hydrolysis where a water molecule or hydroxyl residue initiates the first step leading to the release of a linear intron RNA molecule [47]; the second step is the same as for the branching pathway (Figure 4B). 

In addition to canonical splicing, a peculiar backsplicing reaction observed in various intron types, sometimes generates a class of circular RNAs (circRNAs) in diverse eukaryotic species [104,105]. Two auto-catalytic complex Group I introns that are harbored by the mitochondrial genomes of mushroom corals (Corallimorpharia) and one of these introns is processed by back-splicing [106]. It has been suggested that in the latter example, backsplicing could generate transcripts more suitable for the expression of intron encoded ORFs [106].

Another variation of “standard” intron splicing is trans-splicing [27]. Trans-splicing allows for fragmented genes (gene segments transcribed from different loci) to be assembled at the RNA level as intron components flanking the separated exons can assemble in trans and, therefore, facilitate the joining of the flanking exons generating a contiguous transcript [107,108,109]. Plant mitochondria, in particular among the angiosperms, and to a lesser degree in the gymnosperms, have trans-split genes in which rearrangements have occurred due to various recombination events within Group II introns [110,111] so that exons (and flanking half-introns) are dispersed within the genome, independently transcribed and the mRNA is generated through splicing in trans by assembling the intron components into the proper intron fold [112]. Trans-splicing of organellar introns can be associated with the requirement of RNA editing and multiple protein co-factors [113,114].

In a study of the model Group II intron L1.LtrB from *Lactococcus lactis*, it was reported that the intron RNA can reverse splice into cellular RNAs by reverse splicing utilizing EBS–IBS interactions and the ectopically integrated intron RNAs can interact with upstream alternative circularization sites located on the intron-interrupted mRNA these promote intergenic trans-splicing products, such as novel Group II intron splicing products with mRNA fragments inserted at the spice-junctions. These might provide new functions and mechanisms of generating genetic diversity [115]. Although this is not an organellar intron example it illustrates the versatility and potentially adaptability of Group II intron RNAs in promoting genetic diversity.

## 5. Intron-Encoded Proteins in Guiding Intron Mobility and Multifunctional Roles

**Figure 5:**
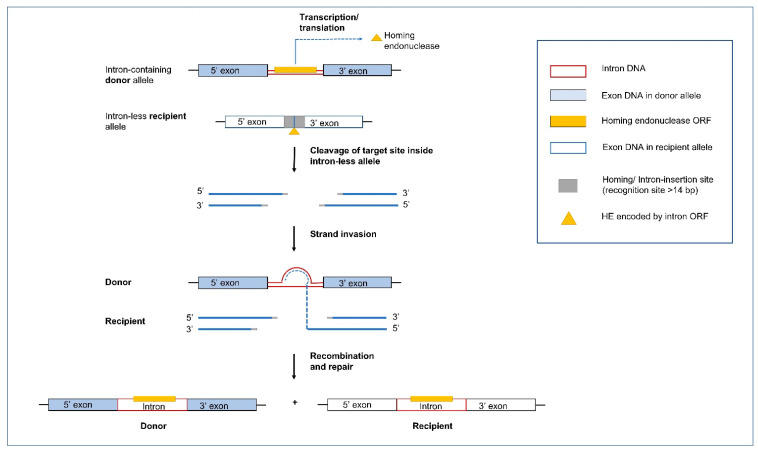
Mobility pathway of the Group I introns through homing. Intron homing is mediated by homing endonucleases (HE, yellow triangle) encoded by the HE-ORF (yellow box) in the intron-containing donor allele. Homing endonuclease target sites (grey box) are typically centered on or near the intron-insertion site in the intron-less recipient allele and include DNA sequences both upstream and downstream of the intron insertion site (usually > 14 bp). The HE introduces a double stranded break (DSB) or a single stranded nick in the intron-less allele. This DSB or nick are repaired by recombination based DSB repair pathway that uses the intron containing allele as a donor template. The end result is the non-reciprocal transfer of the mobile intron element into the intron-less allele.

**Figure 6:**
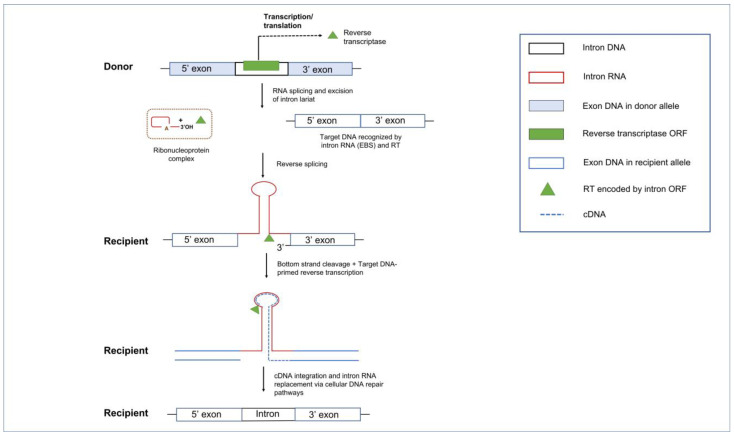
Mobility pathway of Group II introns through retrohoming. Intron retrohoming is mediated by the multi-functional reverse transcriptases (RT, green triangle) encoded by the RT-ORF (green box) in the intron-containing donor allele. The RT protein binds to the intron resulting in a ribonucleoprotein (RNP) complex (brown dashed box) containing the excised intron lariat RNA (red) and the tightly bound RT. RNPs recognize the DNA target by using both the RT (DNA binding domain) and base-pairing of the intron RNA (via the EBS) and then promotes reverse splicing of the intron RNA into the top strand of the dsDNA. After reverse splicing, the bottom DNA strand at the target site is cleaved by the endonuclease domain of the RT. The 3′ end generated at the cleavage site is used as a primer for target DNA-primed reverse transcription of the inserted intron RNA. The original intron RNA (inserted in the sense or top strand) and the resulting intron RNA derived cDNA (blue dashed line) will be integrated into the host genome by the host DNA recombination or repair mechanisms.

Group I and Group II introns are commonly referred to as ‘mobile introns’ as they can move from an intron containing allele to a cognate intron-less allele; this is sometimes referred to as “homing”, or “retrohoming” for Group II introns (Figure 5 and Figure 6). Mobility can be attributed to the presence of IEPs, such as HEs for Group I introns and RTs for Group II introns. The IEPs for both types of introns have also been associated with maturase activities that facilitate (or enhance) the autocatalytic properties of these elements at the RNA level to meet the cellular demand for rapid transcript maturation and RNA turnover. Maturases are assumed to bind to intron RNAs and promote the formation or stability of splicing competent RNA folds [116,117,118,119]. 

Historically, the link between the mobility of Group I introns and the intron encoded HEs was determined by research on the “omega” intron (mitochondrial DNA *rnl* intron) in yeast [120,121,122,123]. From these pioneering studies till now, it has been shown that HEs are usually cis-acting and target-specific with some minor variability permitted in their homing or cleavage site an adaptation to prevent being eliminated due to neutral mutations at their target sites. Indeed, it has been recorded that the HE sequence-degeneracy usually corresponds to wobble positions within the reading frames of protein-coding host genes at the HE recognition site [124,125,126]. 

### 5.1. Group I Introns and Homing Endonucleases

Homing is initiated by HEs that are site-specific DNA endonucleases that recognize a specific target sequence in an intron-less allele, ranging from 14 to 44 bp in length [127]. HEs are typically cis-acting by catalyzing the mobility of the genetic element that encode them. HEs introduce a double-stranded break (DSB), in the intron-less alleles triggering the host DSB-repair or synthesis-dependent strand annealing mechanism utilizing the intron/containing allele as a template to repair the break in the recipient intron-less allele. The end result is the non-reciprocal transfer of the intron-containing composite element (intron including the HE gene or HEG) into the intron-less allele [33,35]. In some instances, regions flanking the introns are also moved along to the new site along with the actual intron sequence due to gene conversion.

There are currently six recognized classes of HEs, named after the conserved amino acid motifs: LAGLIDADG, H-N-H, His-Cys box, GIY-YIG, PD-(D/E)xK, and EDxHD [128]. In fungal mitogenomes, the most abundant families are the LAGLIDADG and GIY-YIG HEs. In addition to these known classes, there are probably more classes yet to be characterized. For example, F-CphI encoded by ORF117 of cyanophage S-PM2 to represent a new HE family, defined by the motif DHHRN [129]. Another novel example of a HE derived element is the yeast mating-type switching endonuclease HO, which has become a domesticated member of an unorthodox homing genetic element family and it deviates from the classical definition of a HE in the sense that it does not propagate its own DNA sequence (does not ‘home’) and has rather been adapted for other normal cellular functions [130].

Some IEPs encoded by Group I introns have dual maturase and HE activity and some HEs may have lost their DNA cutting activity and only act as maturases [131]. It has been reported that HEGs can move independently from their ribozyme partners [132], although recent studies suggest that intron encoded HEGs co-evolved with their ribozyme partners [133,134].

Among the organellar genomes, the LAGLIDADG HE family is most frequently encountered. These enzymes can have one or two LAGLIDADG domains. The single motif LAGLIDADGs are active as homodimers and recognize sites that are palindromic in nature (18–22 bp with recognition flexibility). In contrast, double motif LAGLIDADGs are active as monomers and are not restricted to palindromic DNA target sequences allowing these elements to be more flexible in adapting to new target sites [12,134,135]. Usually, LAGLIDADG HEs cut at their DNA binding site. The second most abundant HE family consists of the GIY endonucleases, characterized by a conserved amino acid motif GIY-(X_10–11_)-YIG and these act as monomers that tend to cut upstream of their DNA binding site [136]. These HEs, like the LADGLIDADGs, can be free-standing ORFs but may also be located within mobile Group I introns in the fungal mitochondrial DNAs, in algae, and in the chloroplast DNA of plants [137].

The coevolution of fungal mitochondrial introns and their corresponding HEs (GIY-YIG and LAGLIDADG families) has recently been studied to propose a model called the “aenaon hypothesis” which describes the invasion of ancestral free-standing HEGs into introns through the evolutionary events of recombination, transposition, and HGT [133]. The coevolution of the HEGs associated with introns is crucial in understanding the variability in the organellar genomes. It has been observed that frequently intron encoded ORFs are fused to the upstream exons of their host genes. The inter-dependence of LAGLIDADG and GIY HEs with their upstream exons provides these ORFs access to the cis genetic elements that are required for their expression [138,139]. The “aenaon model” combines characteristics of the “intron late” and “intron early” theories [4], proposing that there are ancestral introns and HEGs with conserved site recognition throughout fungal evolution. Mitochondrial introns have evolved bidirectionally, either, through migration to similar target sites but with different actual locations (ectopic, for example, orthologous introns associated with *cox1* and *cox2* genes) or through change in the intron RNA structure of their compact ancestral form (expansion through addition of new hairpins or the less common, reverse reduction). The expansion or reduction in the RNA structure of the introns themselves, is accompanied by the change in the overall architecture of the mitochondrial genomes, with the persistence of certain ancestral intron forms along with the addition of new introns in the mitogenomes of higher fungi. Over time, free-standing HEGs have also been replaced with intron-associated HEs [140]. Recent studies have shown that for LAGLIDADGs, minor amino acid changes within the HEs active sites can cause the HE to target different sites, thus allowing the HE plus their ribozyme host to transpose to new sites [12]. The “aenaon model” proposes a dynamic coevolution pattern of introns and HEGs, resulting in the great diversity of intron complements as observed among the fungal organellar genomes [141,142]. These findings may also be relevant to non-fungal organellar genomes. 

Phages have proven to be useful model systems for studying the biology of HEs including insights on the evolution of composite introns whereby a ribozyme gained an ORF that could promote its mobility [139,143]. Among phages, stand-alone HEs are observed frequently and these HEs can promote their own mobility. However, there are instances where introns (without ORFs) insert into homing sites that are also targeted by free-standing HEGs. 

Studies on phage introns and HEGs support the concept that introns and their HEGs evolved independently as both target conserved genes for their survival, those introns and HEGs targeting orthologous sequences eventually merged into composite mobile elements [29,129]. These arrangements would be mutually beneficial reducing competition for the same target site by the two independent elements and once merged, the intron has a “built in” mobility promoting catalyst and the HEG occupies a “neutral location” minimizing its impact on the host genome. 

LAGLIDADG HEGs can be components of Group I and less frequently of Group II introns [46,131]. HEGs can move along with their host introns or they can self-propagate within and in between genomes [143]. A GIY-YIG type endonuclease has also been found to be encoded within a Group II intron [13,144]. A LAGLIDADG HE encoded by a Group II intron located within the *rns* in the filamentous fungus *Leptographium truncatum* demonstrated to have the capacity of provide a means of mobility for its host introns but did not display any maturase activity [145]. This study provided the first biochemical analysis of a Group II intron that encodes a Group I intron derived LAGLIDADG HE rather than a RT. Intron homing dependent on a Group-II intron encoded HE was also studied in the maize smut fungus, *Ustilago maydis* [146]. This study focused on a Group II intron located within the *rnl* gene. The intron encoded HE (I-*Uma*I) represents a Mg^2+^-dependent endonuclease that requires both LAGLIDADG domains for activity and recognizes a minimum target site of 14 bp and can provide the Group II intron with a DNA based homing mechanism. 

HEGs are viewed to be diversity generating elements and can propagate efficiently in organellar genomes by a homing mechanism that is based on homologous recombination. Their site-specific nature favors targeting conserved sequences and they persist in the presence of repetitive genes or genomes (multi-copy) and in organisms where there are opportunities for vertical and horizontal transmission of genetic elements [59,147]. For most cytoplasmic components (including mitochondria and chloroplasts), inheritance tends to be uniparental, ensuring uniparental inheritance of HEGs and their intron partners. However, so-called “super-mendelian” inheritance has been noted in uni- or bi-parental inheritance systems in yeasts where introns can spread to the progeny efficiently due to the potential of mitochondria originating from the two mating types fusing and introns homing into intron-less alleles [123]. HEs catalyze intron mobility in organelles and there are indications that nuclear genes may impact the mobility and possible inheritance of mobile introns. A recent study on the impact of the nuclear gene *SXI1α* in the *Cryptococcus neoformans* species complex on mitochondrial inheritance and intron/HEG transmission showed that disruption of this gene results in biparental mitochondrial inheritance and significant heteroplasmy and an increase in the number of introns in the progeny. Thus, this sex-determining nuclear gene *SXI1α* appears to be critical for inhibition of the spread of HEGs in the mitochondrial genome and its absence leads to the over-transmission of HEG-containing introns [147]. This study shows that there are mechanisms that can modulate the impact (or number) of organellar mobile elements.

### 5.2. Group II Introns and Reverse Transcriptases

Mobile Group II introns spread within and between genomes by a mechanism of “retrohoming” in which the intron RNA inserts directly into a DNA site and is reverse-transcribed by an intron-encoded RT [148]. Group II intron encoded proteins are multifunctional due to the presence of several protein domains: the N-terminal RT domain followed by the X (or maturase) domain. Some RTs also contain a DNA-binding domain (DBD) and an endonuclease (EN) domain at the C-terminus of the RT protein [47]. 

In the retrohoming pathway, the basic steps include the release of the intron RNA lariat from the transcript followed by the intron RNA binding of the intron encoded RT protein to form a ribonucleoprotein (RNP) complex which can initiate retrohoming. The RNP complex can scan for the DNA target site utilizing the RT DBD and the EBS segments on the intron RNA. The EBS elements guide the RNP to a homing site via base pair complementarity and the 3′ end of the intron lariat can generate a single stranded cut at the double stranded DNA target site. This single stranded cut allows for the process of reverse splicing of the intron RNA into the DNA homing site. For those RTs that have an EN domain, the RT will cleave the complementary strand of the target site; this will generate a 3′ end that can be utilized as a priming site for the RT to start the reverse transcription of the intron RNA into cDNA [149,150,151,152,153]. The intron RNA will eventually be replaced by the DNA repair system. Retrohoming involves numerous host factors, such as components of the DNA repair and recombination machinery, and DNA replication components [45,154,155,156,157].

Group II introns have diversified the genomes in all domains of life, and they are good candidates for being the progenitors of the eukaryotic spliceosomal introns (see Figure 1). Evidence for their common ancestry is apparent from similarities between recently resolved crystallography-based structures of the eukaryotic spliceosome and structures of Group II intron IEPs and selected Group II intron RNAs. Shared features in RNA secondary structural elements, metal ion binding sites, and parallels in splicing mechanism have been discussed extensively in previous reviews [158,159,160,161,162]. Two major events would have facilitated the transformation of Group II introns (or a common ancestor) into spliceosomes. The first would be fragmentation of a Group II intron RNA into pieces (such as the bipartite and tripartite Group II introns observed in plants), ultimately resulting in functional RNAs that would operate on splice sites in trans. In addition, the recruitment of splicing factors occurred to compensate for the loss of RNA structural features that optimized autocatalysis of the splicing reaction and to facilitate splicing reactions in trans-configurations. Finally, which is mechanistically dependent on the first, would be the ability of Group II intron components to function as multiple-turnover enzymes that catalyze splicing at numerous sites. One interesting finding is the domestication of the Group II intron encoded RT into splicing factors such as the nuclear protein PrP8 which is an important component of the spliceosomal machinery involved in removing nuclear spliceosomal introns [163,164,165]. Other RT derived splicing factors are the nuclear encoded nMAT1, nMAT2, nMAT3, nMAT4 that facilitate splicing of introns in the mitochondrial genomes of the angiosperms [166,167], and matR (located in plant mitochondrial genomes) and matK (located in plant chloroplast genomes) [168].

### 5.3. Maturase and Splicing Factors

As stated previously, early research indicated that under cellular conditions, most so-called self-splicing introns require intron or host genome encoded protein factors for efficient splicing [69,169,170,171,172]. Some factors are intron encoded (maturases) and act on their own intron RNA facilitating RNA folding into splicing competent configurations. Intron encoded HEs have been suspected to be potentially bi-functional serving as maturases and as catalysts for mobility [131,173] and Group II intron encoded RTs have a maturase (X) domain that promotes intron splicing [24]. Additional host genome encoded factors are either moonlighting (i.e., acquired a second function) or have been co-opted to promote the splicing of organellar introns. These splicing factors compensate for the loss of functional IEPs in many Group II introns and for changes or mutations in the intron RNA sequences/structures that occurred during the evolution of these introns that are required for splicing.

Historically the connection between nuclear genes and mitochondrial intron splicing was discovered by analyzing respiratory mutants in *Neurospora crassa* and *Saccharomyces cerevisiae* [174,175,176]. In *N. crassa* the mitochondrial tyrosyl-tRNA synthetase (nuclear encoded CYT-18 protein) promotes the splicing of Group I introns. In addition, in *N. crassa* nuclear encoded proteins such as the DEAD box RNA helicase protein (CYT-19 protein) has been shown to be required for splicing Group I and II introns [177,178,179,180,181] and the CYT-4 protein (a protein with an exoribonuclease 3′ to 5′ domain) is involved in Group I intron splicing [182,183]. In *S. cerevisiae* similar nuclear encoded proteins have been identified (*Mss116* (homolog of CYT-19)- DEAD box protein/RNA helicase and chaperone; *NAM2*-leucyl-tRNA synthetase; *DSS1*-putative exoribonuclease) [184,185] that are required for the efficient removal of mitochondrial introns. The DEAD-box helicase Mss116 is a multifunctional protein that has been demonstrated to be an RNA chaperone and by interacting with other cofactors it is involved in organellar intron splicing, mitochondrial ribogenesis, and a factor needed for the translation of some mRNAs [186]. There are probably many more proteins that are involved in promoting organellar intron splicing, thus, contributing towards the crosstalk between the nuclear and organellar genomes and examples like Mss116 show the connection between various aspects of organellar RNA transactions. 

Plant mitochondrial and chloroplast organelle introns also require intron and nuclear genome encoded protein factors for their splicing. Group II intron encoded proteins (RT-like proteins) have maturase domains [103]. Many plant organellar introns have deviated from their ancestral RNA structures and many do not have ORFs, so it is believed that there is a high requirement for maturases and various co-factors to compensate for these evolutionary changes [68,69,187]. Plants have many nuclear genes that are involved in organellar intron splicing. For example, the plant organellar RNA recognition (PORR) protein domain family has an RNA binding domain and is found in most members of this protein family that are involved in splicing of organellar introns [79]. As stated previously, there are nuclear (nMAT1 to 4) and organellar encoded proteins (matK and matR) that have been derived from Group II intron encoded reverse transcriptases [77,168]. IEPs typically are assumed to be cis-acting (only assist their native intron) but Group II intron-encoded proteins, such as MatK and MatR and the nuclear nMAT1 to 4, appear to be trans-acting splicing factors that can assist several organellar introns in their splicing activities [167,188,189,190]. Another example of a putative intron IEP derived nuclear encoded splicing factor has been characterized in *Arabidopsis thaliana* [191], the OTP51 encoding gene. This nuclear gene expresses a protein that has the pentatricopeptide repeat (PPR) RNA binding domain plus two LAGLIDADG motifs and this protein is required for the cis-splicing of a chloroplast Group II intron. LAGLIDADG motifs are frequently encountered in Group I intron IEPs and have been associated with maturase activity promoting the splicing of Group I introns. 

Among the plant mitochondria, one frequently encounters trans-spliced introns where two initially separate transcripts become covalently joined through the splicing process. The mechanism of mitochondrial intron splicing is complex involving many splicing factors, which facilitate the trans-splicing of Group II introns, including pentatricopeptide repeat (PPR) proteins, chloroplast RNA splicing and ribosome maturation (CRM) proteins, RNA DEAD-box helicases, plant organellar RNA recognition (PORR) domain proteins, regulator of chromosome condensation (RCC) proteins, and others [192,193,194,195]. Among them, PPR, PORR, and RCC may act as the factors that recognize the specific RNA-binding sites [69]. 

RNA editing is a feature of organellar gene expression and is linked in some cases to intron splicing. One example demonstrating the link of RNA editing and splicing is the chloroplast RNA helicase increased size exclusion 2 (ISE2) in *Arabidopsis,* which is a chloroplast localized nucleus-encoded splicing factor for Group II introns. ISE2 is evolutionarily conserved in all photosynthetic organisms including cyanobacteria, and, along with other helicases, form the ‘editosome’ which can access its substrates once ISE2 unwinds the local RNA secondary structure, and it also acts to remove the complex on completion of RNA editing, and promote annealing of edited RNA [196]. Loss of ISE2 results in defects in C-to-U RNA editing, altered accumulation of chloroplast transcripts and chloroplast-encoded proteins and defective processing of chloroplast ribosomal RNAs. This altered accumulation of chloroplast RNAs encoding for the photosynthetic machinery indicates that ISE2 may either act at the transcriptional or post-transcriptional level to modify chloroplast transcription or RNA stability.

The impact of organellar intron splicing and protein factors that impact splicing are further illustrated in *Arabidopsis*, where mitochondrial intron splicing, and overall respiratory functioning, especially during stress response reactions, depends on the CFM9 protein, which is a mitochondrial chloroplast RNA splicing and ribosome maturation (CRM)-domain containing protein. A study on splicing efficiency (ratio of spliced to unspliced transcripts) between *cfm9* mutant and wild-type protein showed, not only decreased transcript accumulation for several *nad* gene associated introns, but also resulted in developmental defects, such as delayed seed germination, retarded growth, and shorter height, especially when subjected to salinity and dehydration stress [197]. In maize, the pentatricopeptide protein EMPTY PERICARP 8 is involved in the splicing of Group II introns in the mitochondria, complex I biogenesis and seed development. The loss-of-function *emp8* mutant is embryo lethal with severely arrested embryo and endosperm development and the impairment in respiration and loss of activity of the mitochondrial complex I is caused by splicing defects (trans-splicing of *nad1* intron 4 and cis-splicing of *nad4* intron 1 are abolished; cis-splicing of *nad2* intron 1 is severely impaired). These results highlight the importance of EMP8 in mitochondrial functions, seed development and as a key member of a putative “mitospliceosome” involved in intron splicing, along with other PPR proteins [198]. Another example is the nuclear *Empty pericarp 11* (*Emp11*) gene that encodes a PPR protein in maize and this protein is crucial for *nad1* intron splicing. It is involved in the recognition of the precursor *nad1* transcript and for maintenance of the *nad1* RNA fold conformation for intron splicing. Loss of function mutants show impaired development with empty pericarps and reduction in mitochondrial complex I assembly and activity, structural disturbances, and increase in alternate oxidase activity (indicative of a disruption in the electron transport chain). This shows that retrograde signaling between mitochondrial and nucleus gene expression ensures the integrity of the mitochondrial intron splicing machinery [199]. One has to assume that a similar situation exists for chloroplast integrity and function and nuclear genome components that regulate the expression of plastid genes including the orderly splicing of introns [114].

## 6. Introns: At the Fulcrum of Adaptation and Elimination 

The ability of an intron to transpose to new sites might rely on the acquisition of novel target recognition sequences through accumulation of mutations in the IEPs. Reverse splicing of intron RNAs into RNA sequences is less efficient as there is a requirement for a reverse transcription step followed by recombination but the RNA interactions (wobble, etc.) might be more relaxed compared to protein mediated mobility. Homing endonuclease interactions with target sites is in part based on indirect readout (i.e., not necessarily based on specific nucleotide bases interacting with specific amino acids) so sometimes minor changes at the protein level can shift the enzymes specificity to a new target site [12,200]. However, once a mobile intron has inserted into a new target site its persistence is based on drift (neutral evolution) and natural selection [134,201]. If the element is neutral with regard to its impact on the host genome it can accumulate mutations leading to degeneration of the IEP coding and ribozymes sequences leading to the loss of the intron. Maintenance of an intron is dependent on the correlation between the energy burden of housing the intron and possible advantages the intron may provide [202].

Goddard and Burt (1999) examined the distribution of HEGs and their host introns in members of the Saccharomycetales [203]. They noticed that HEGs along with their intron partner move into a target site, but this is followed by the accumulation of mutations in the HE ORF sequence leading to the loss of the ORF and eventually the intron sequence is completely lost. This re-establishes the site for an orthologous HEG to reinvade this location. These observations were formulated into the HE lifecycle where mobile introns are neutral elements that, due to lack of selection, rapidly accumulate mutations, and degenerate. They can persist by outpacing drift at their sequence level by moving into empty target sites or invading new sites. The persistence within populations requires the opportunity for outcrossing, horizontal and vertical transfers, and cytoplasmic transmissions (such as heterokaryon formation). The pattern of rapid spread through a population followed by degeneration may be a common property of introns and other selfish genetic elements due to a lack of selection, however this balance can shift if introns provide benefits, such as acting as regulatory elements that can modulate gene expression, encoding essential proteins, or having been co-opted to provide essential functions. Intron conservation across some plant, metazoan, protozoan, and fungal organellar genomes would suggest that introns may not always be merely neutral elements. Trans-splicing introns might be a more permanent feature that allows fragmented genes to be assembled in trans at the RNA level. Introns encoding splicing factors (i.e., matK and matR) that can act in trans are also integral components of organellar gene expression.

**Figure 7:**
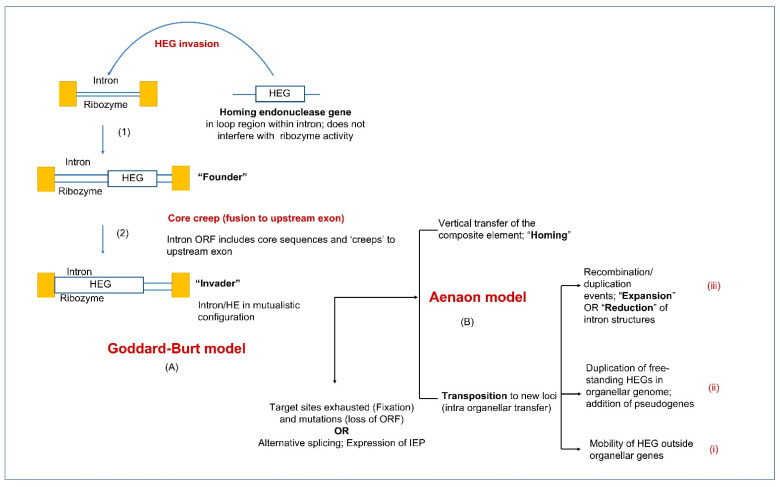
Comparison of the Goddard–Burt (as modified in [138]; copyright license number 5111000556510) and the aenaon models of intron/HEG evolution. In the first step, the HEG invades and resides within the intron and does not interfere with its catalytic activity. This element is called the ‘founder’ mobile intron. Intron ORFs, over time, expand and the ORF includes intron core sequences and creeps towards the upstream exon by ‘core creep’. The result is fusion of the intron ORF to the upstream exon. In the ‘invader stage’, the intron/HE combination has established a mutualistic configuration that minimizes the impact on the host gene and favors the expression of the IEP which would increase the probability of intron mobility and efficient intron splicing [138]. As suggested by Goddard and Burt, once a mobile intron has exhausted its target site and undergone fixation, accumulation of introns may lead to the loss of the intron ORF fused to the upstream exon [203]. This cycle ties into the aenaon model [133], to explain the fates of the introns on homing and transposition both at inter and intra-organellar levels. Upon transposition to new sites within the organelle, the composite intron-HEG element can (i) show mobility outside the organellar genes; (ii) free-standing HEGs can undergo duplication within the organellar genome, with probable addition of pseudogenes due to drift; (iii) genetic recombination and duplication can lead to either expansion or reduction in intron RNA structures.

The original concept of Goddard and Burt’s (1999) HE lifecycle has also been applied to all types of mobile introns and has been modified as more organellar genomes have been characterized with regard to their intron contents. It is assumed that ribozyme-type introns originally existed as self-splicing introns that, in some instances, acquired ORFs. In the case of Group I introns, HEs provide a means of more efficient mobility. In turn, HEs evolved maturase activity to promote efficient splicing ensuring the composite element minimizes its impact to the host genome [170]. Composite introns as predicted by Goddard and Burt (1999) can be gained and lost and regained due to drift. However, there are other features of complex introns that are worth noting. Many intron sequences that encode proteins are fused to the upstream exons of their host genes. This has been referred to as ‘core creep’ where the intron ORF over time has incorporated upstream intronic sequences to fuse in-frame to the upstream exon [204]. This fusion would allow the intron encoded protein to be more efficiently expressed, as it gains regulatory sequences of the host gene that optimize translation [138,204]. The cyclical model representing the intron life cycle has now been modified to include ‘escape routes’ wherein introns escaping from this cycle can gain new biological functions and subsequently, novel RNA elements with roles beyond splicing have originated (see Figure 7). The concept of core creep is also supported by the observation that there is a bias of mitochondrial introns towards being so-called phase 0 introns (i.e., they do not interrupt codons) [142,205]. Intron insertion sites at phase 0 facilitate the fusion of intronic ORFs in-frame with the upstream exon. Composite introns can expand by gaining additional ORFs or intron modules [138], and there are instances of internal (non-fused) ORFs being expressed via AS that generates a product that fuses the intron ORF sequence to the upstream exon. This has been referred to splicing mediated core creep [138,142] and it demonstrates the complexity of the intron ORFs co-evolving with their intron host sequences and the host gene sequences for optimizing the expression of IEP and minimizing their impact on the host genomes. It has been suggested that the intron ORFs are essentially mutualistic towards their host introns and some IEPs may impact the persistence of other introns (reviewed in [141]) suggesting that drift is only one component that may explain the persistence and distribution of organellar introns.

## 7. Introns Impacting Gene and Phenotypic Expression

Organellar introns can be beneficial, as they encode proteins such as ribosomal proteins (rps3), aminotransferases, and N-acetyltransferases and trans-acting maturases [168,206]. In fungi, mitochondrial introns have been associated with fungicide resistance, a recent example being Group I-D introns associated with the *cytochrome b (cob*) genes in the mitogenomes of 169 fungal species studied by Cinget and Bélanger (2020) [207]. Previously Grasso et al. (2006) have hypothesized that the presence of a *cob* Group I mitochondrial intron blocked the mutation involved in the resistance against quinone outside inhibitors (QoI) fungicides [208]. Presence of Group I introns in certain positions within the *cob* gene of fungi appeared to prevent mutations in the flanking exons in order to maintain sequences required for the P1 and P10 interactions that are required for intron splicing. This sets a constraint on mutations arising that are responsible for fungicide resistance. Overall, the correlation between this resistance phenotype and the Group I intron is dependent on the presence of compatible homing sites in the mitogenomes and transient displacement of this intron [207]. 

The presence of an ORF-less Group II A1 intron inserted in the *rns* gene of the chestnut-blight fungus *Cryphonectria parasitica* appears to induce hypovirulence [209]. This study showed that this intron spliced inefficiently resulting in a low production of mitochondrial ribosomes. In addition, it was demonstrated that the attenuated-virulence trait and the splicing-defective intron can be transferred asexually via hyphal contact from hypovirulent (intron) donor strains to virulent recipients. Hypovirulence results in the fungus infecting its host tree without causing detrimental consequences to the host. The attenuation of virulence is beneficial for the pathogen population as it prevents the extinction of the host species which, in turn, prevents the extinction of the pathogen.

Introns in general have been viewed as possible vehicles for regulating gene expression, as their removal can be a rate limiting step for the expression of the genes that contain them [210]. A study on yeast showed that the persistence of self-splicing mitochondrial introns is facilitated by an evolutionary lock-in, wherein the host genome has adapted to primordial invasion of introns in a way that subsequent intron loss could be deleterious [211]. The fitness of intron-less yeast strains was compared with the wild-type intron rich strains and it was found that the strain without mitochondrial introns has altered mitochondrial morphology, gene expression, and metabolism impacting its growth and life span. Another example of introns serving as regulatory genetic elements, comes from the hexacoral mitochondrial Group I introns associated with the *nad5* and *cox1* genes. Here, the introns appear to have been domesticated and gained novel host-specific functions beyond self-splicing [212]. 

Another dimension of self-splicing introns has been demonstrated in bacterial systems. Studies suggest that mobile self-splicing elements might have evolved opportunistically in sync with their hosts, contributing to cellular stress response pathways [213]. There has also been a recent report on the intrinsic cold sensitivity of the pRS01 plasmid-encoded *Lactococcus lactis* Group II intron, Ll.LtrB and its IEP that could be involved in cold stress adaptation [214]. In *Clostridium difficale,* one example of conditional RNA splicing is dependent on a Group I intron derived riboswitch that is dependent on the absence or presence of cyclic GMP causing the riboswitch to assume differing configurations generating splice variants that can determine if the mRNA is translated or not [215]. Group II intron splicing can also be affected by conditional cues, an example being the *E. coli* LL.LtrB Group IIA intron which is stimulated by intra- and extracellular Mg^2+^ concentrations [216]. Although these are not organellar examples they may be indicative that some Group I and II introns and their IEPs might have co-evolved with their host genomes or have been co-opted as genetic elements that can respond to environmental conditions that potentially benefit both the host and the mobile elements.

Evolutionary process is an interplay between selection of mutations and random drift, where selection can occur only when the drift barrier is overcome (the drift barrier is denoted by the product *N_e_ s*, where *N_e_* is the effective population size and *s* is the selection coefficient associated with the given mutation) [217,218,219,220]. Population-genetic studies indicate that, taking into account the characteristic values of *N_e_*, mutation rate and the target size for deleterious mutations in splicing signals (only about 25 bp/intron), purifying selection in typical populations of multicellular eukaryotes is too weak to eliminate introns, which have persisted in the eukaryotic genomes since the invasion of mobile elements in the early stages of eukaryotic organellar evolution [221]. Sequences originating from mobile genetic elements are frequently recruited into essential eukaryotic genetic elements and proteins, snRNAs and spliceosomal introns, components of promoters and enhancers, and components of riboswitches. An example is the Group II intron encoded IEP that evolved into telomerase and the essential spliceosomal subunit Prp8 protein [165,222]. 

## 8. Evolutionary Spread of Mobile Organellar Introns across the Three Domains and Organelles

Over the course of evolution, several genetic elements have moved by horizontal transfer between chloroplast and mitochondrial genomes of heterokonts with recombinases likely promoting such gene exchange events. For example, in diatoms, there is a sporadic distribution of mitochondrial introns which is assumed to be the result of high rates of intron loss during evolution [223,224,225]. Such patchy distributions are often, in part, the consequence of HGT, which makes it difficult to distinguish recent gains via HGT from widespread losses of an ancestral feature [225,226,227,228,229]. Guillory et al. (2018) sequenced mitochondrial genomes from five diverse diatoms and found that the vast majority of the introns were Group II type and situated in the mitochondrial genes *cox1* and *rnl* [230]. They reported through phylogenetic studies, that they arose out of the ancestral heterokonts (aquatic flagellate eukaryotes), mainly algae, with intron-rich mitochondrial genomes, through intron losses and horizontal transfer. Diatoms gained their chloroplasts by secondary endosymbiosis, i.e., the incorporation of a eukaryotic endosymbiont, such as red or green algae [231], setting the stage for gaining various genetic elements from this endosymbiont. HGT can also be mediated by other mechanisms that may involve viruses and as of yet unknown DNA uptake mechanisms.

Another example of a probable HGT is in the diatom *Toxarium undulatum* where a large Group IIB intron in the *psaA* gene may have been introduced into this plastid genome from an unknown donor [224]. This is the first report of this novel Group II intron in diatom plastid genomes and indicates a shared history with green algal introns [232,233]. Sequencing of the chloroplast genome of the pennate diatom *Seminavis robusta* reported the first instance of the presence of two introns in the chloroplast genome, one being a Group I intron in the *rnl* gene, encoding a putative LAGLIDADG HE and the second one being a Group II intron in the ATP synthase subunit beta *(atpB*) gene encoding an RT closely related to an RT in the chloroplast genome of the green alga *Volvox carteri.* This supports the theory that the enlarged size of the diatom chloroplast genome may have been because of various instances of horizontal transfer (through homing of introns, recombination and other HGT mechanisms) and the recent arrival of the *atpB* intron in this genome from the *Volvox*-like alga [234]. Mitochondrial Group II introns associated with the *cox* genes in the heterokont protozoan *Chatonella* spp. present an example of lateral transfer from diatoms to rhodophycean dinoflagellates [227]. Diatoms are an important component of plankton which comprises many eukaryotes that are mixotrophism surviving by photosynthesis in combination with ingesting prey providing opportunities for HGT.

The characterization of the mitochondrial genome of a heterotrophic unicellular member of the Centrohelea, *Marophrys* sp. strain SRT127 showed evidence of intron transfer between the mitochondrion and green algal plastids, and also showed the integration of a linear invertron-type plasmid into its mitogenome [235]. Members of *Marophrys* do prey on algae and this may have facilitated the transfer of genetic elements from green algae to the predator organellar genome. As stated in previous examples, organisms co-existing in close proximity ether by forming symbiotic or predator–prey relationships are more prone to HGT.

In plants, the rates of HGT in the two organellar genomes, mitochondria and plastids, are strikingly different, possibly because of more efficient uptake of exogenous DNA by the mitochondria and greater propensity of donor and recipient mitochondrial fusion compared to plastids [236]. The most frequent case of horizontal transfer in plants, involves a *cox1* Group I intron and it was recently acquired by three lineages of the angiosperm family the Solanaceae, through a within-family transfer event, indicate by the identity of intron sequences and the sequence of the flanking exons (co-conversion tract downstream of the intron) [237]. Parasitic plants are often involved as donors or recipients in the horizontal acquisition of mitochondrial genes, an example being a single HGT event involving a strictly DNA-level intermediate resulting in the transfer of *atp1*, *atp6,* and *matR* from the parasitic *Cuscuta* (Convolvulaceae) to at least three species of *Plantago* (*P. coronopus, P. macrorhiza,* and *P. subspathulata*) [238]. It is also interesting to note the events following HGT in plants, wherein, after a duplicative HGT phenomenon (integration of a foreign region apart from a native homologous locus) massive recombination of foreign and native loci occurs, leading to a lineage of diverse, differentially mosaic genes. This has been found in members of Ericales (for example, *Ternstroemia* sp.), which presented models of intramitochondrial retroprocessing and interorganellar gene conversion [239]. These examples show that HGT involving the mitochondria is more frequent in plants, and there are only a few reports of mitochondria-to-chloroplast HGT as the chloroplast genome lacks an active DNA-uptake system. One case of a mitochondria-to-chloroplast HGT event can be seen in the transfer of the *rps16* gene of mitochondrial origin in chloroplast genomes of most of the higher plants [240].

HGT between plants and fungal organelles was first demonstrated by the occurrence of a *cox1* Group I intron in the vascular plant, *Peperomia polybotrya* [241]. More recently, sequential HGT events have been noted for the ancestors of the orchid subfamily, Epidendroideae, which were obligate parasites on Basidiomycete fungi during early development. Unlike the previously documented HGT event between fungi and plants which involved transfer of a homing intron, this study documents the horizontal transfer of sequence comprising up to ~8 kbp involving at least a dozen fungal genes remnants, along with their intronic and intergenic regions. Overall, almost a third of the fungal genome was incorporated into the orchid genome, pointing at a genome-scale chimerism between a land plant mitogenome and a fungus [242]. 

A novel mechanism of mitochondrial gene evolution has also been recently investigated, which involves partial foreign gene replacement, which is achieved through intron mobility in *Tremella fuciformis* [243]. In this case, intron mobility associated with gene conversion involving flanking sequences and insertion of this introns resulted in the generation of N-terminal duplication of mitochondrial genes, leading to partial fragment exchange through lateral transfer. Among sponges, the subclass Spirophorina (Tetractinellida) is an intron hotspot and shows the co-occurrence of two introns in *cox1* which represents a combination between an active and a degenerating intron, presenting the scenario of both HGT, and to a lesser extent, vertical gene transfer and secondary loss of introns [244]. 

## 9. Intron Gain and Loss Resulting in Dynamic Organellar Genomes

Organellar genomes in plant and fungi are highly dynamic because of the loss and gain of mobile elements throughout evolution. Intron gain might occur due to homing (Group I) or retrohoming (Group II), and also due to HGT, leading to expanded and inflated genomes. However, there are also instances of streamlining that can be due to drift or selection which might be lineage specific, where the cost of maintaining introns impact fitness.

Among fungi, large-scale genome rearrangements are often accompanied by accumulation of intergenic and intronic sequences, resulting in the increase in mitogenome size [238,245]. For example, the fungus *Endoconidiophora resinifera* boasts of an intron-rich mitochondrial genome due to large number of intron insertions in the *cox1* gene, partially justified by the probable benefit of introns as gene regulators contributing to their persistence within a population [141]. Large-scale intron loss events were also recently reported among mitogenomes of ectomycorrhizal fungi belonging to the genus *Boletus*, which also helped explaining frequent gene position reversal throughout evolution [246]. 

Intron content is highly variable in plant mitochondria. In angiosperms, most of the variation is not caused by intron loss, but by an overall higher frequency of gene loss and presumably transfer to the nucleus. Introns can be lost by precise intron deletions or due to local gene conversion after an intron-lacking exogenous gene copy is integrated in the mitogenome by horizontal transfer [247]. Loss of introns is commonly explained by “retroprocessing”. In this model, processed (spliced) mRNA is reverse-transcribed and the resulting intron-free cDNA integrates into the genome by homologous recombination. One of the predictions of the ‘retroprocessing’ model is the positional bias of intron loss towards the 3′ end of the genes, which coincides with the mechanism of reverse transcription that begins at the 3′ end and the RT falls off prematurely from the template before reaching the 5′ end. Another model of intron loss through HGT has been proposed as a more likely explanation of intron loss in angiosperm mitochondria, offsetting the retroprocessing model which is mainly supported by parallel loss of an intron and numerous adjacent RNA editing sites. A study on *Magnolia tripetala* reported that the event of intron loss from the *cox2* gene in the mitogenome cannot be explained by the retroprocessing model due to the sporadic distribution of RNA editing sites. It is more likely that a horizontal transfer event resulted in the intronless gene being acquired from another eudicot and then it underwent recombination and gene conversion with the native intron-containing gene and this study adds an alternative possibility of intron loss mechanism for plant mitogenomes [248].

The dynamics of mitochondrial genome expansion and shrinkage are complex and can substantially vary among members of different genera or among species within the same genus. Extensive variation in content and size is seen among plants and fungi, owing to dynamic gains and losses of repetitive noncoding DNA (intergenic spacers) and selfish genetic elements (introns and transposable elements) that have parasitized these genomes. Such gain and loss events are amplified in organisms with mutualistic lifestyles mainly guided by the need to eliminate potential redundancies and streamline the genome. This is especially applicable to a symbiotic association, such as lichen (an association between a primary mycobiont, an ascomycete fungus and a photobiont, a green alga, or cyanobacterium). Recently, data from 58 lichen mycobionts were used to examine broad-scale patterns of intron gains, losses, and genome streamlining in seven different lineages of lichens: Lecanorales, Peltigerales, Telochistales, Ostropales, Pertusariales, Mycocaliciales, and Arthoniales [249]. Ancestral state reconstruction revealed that 9 species in the dataset had undergone loss of introns in the *cox1* gene along with substantial reduction in intron numbers in all mitochondrial genomes, and intron loss events far exceeded gain events, providing evidence of parallel streamlining of mitochondrial genomes via loss of parasitic elements like HEGs and introns in symbiotic organisms. This study also reports that asexual lichens accumulate introns faster than sexually reproducing taxa, probably due to shorter generation time and accumulation of small deleterious mutations. However, it must be remembered that genome reduction is also accompanied by gains in several lineages and thus the losses cannot be exclusively regarded as a propeller for evolution among lichenized fungi and the dynamic loop of gain and loss affects the event of stable or consistent streamlining. However, another recent report on a lichenized microalga, *Trebouxia* sp. TR9, belonging to Trebouxiophyceae (Chlorophyta) showed that this symbiotic association does not seem to have an impact on mitogenome gene content, which is in contradiction to the usually favored genome reduction in symbiotic relationships. This genome is more comparable to streptophytes, where conservation during evolution from a common ancestor led to maintenance of a large mitogenomes [94,250]. 

In addition to plant and fungal organelles, recurrent loss and horizontal transfer of introns have been noted for diatoms which have sporadic and patchy distribution of mitochondrial introns [230]. Intron gain and loss dynamics were also studied in the mitochondrial *cox1* gene of Scleratinia (Cnidaria, Anthozoa). Molecular dating suggested that intron gain and loss might be corroborated with major extinction events [251]. These correlations indicate that mass extinctions might have provided a selective advantage of respiratory efficiency to intron-less species, allowing them better adaptation in harsh climatic conditions. 

## 10. Building Up the Complexity in Introns

The term ‘twintron’ was initially used to describe Group II introns inserted within other Group II or Group III (derived from Group II introns) introns; later other variations were identified, such as Group II in Group I, Group I within other Group I, and lariat capping twin ribozyme introns [16,211]. The original definition implied that the internal components splice out first, allowing the “outer” components to be spliced together and achieve a splicing competent RNA fold. However, many different types of “nested” intron arrangements exist where splicing of the internal intron is not essential for the splicing of the external intron [252]. It is still important for composite multiple intron-module arrangements to achieve splicing competent folds for its various components [253]. In addition, intron and host genome encoded maturases or splicing factors must be involved in resolving transcripts with complex introns. 

Twintrons were first described from *Euglena* chloroplast DNAs where the *psbF* gene which encodes the β-subunit of cytochrome b-559 contained a Group II intron inserted within the structural domain V of another Group II intron [13]. A sampling of fungal mitochondrial *nad5* genes identified a complex Group I intron with two sets of Group I intron core sequences in the *Annulohypoxylon stygium*, while a second complex intron was observed in the *nad5* gene of *Cryphonectria parasitica,* where a Group I intron core encodes a LAGLIDADG ORF that is interrupted by a Group II intron module [205]. A similar arrangement was previously characterized in the mitochondrial mS1247 intron in the *rns* gene [254] and in the *cytb*-506 intron [138]. It has been speculated that the ORF-less Group II intron, that in all cases was inserted in frame with regard to the LAGLIDADG ORF, could be a regulatory element that determines the expression of the IEP of the resident Group I intron [248,255]. A variety of complex introns have been identified in fungal mitochondrial genomes [138,141,205,254,256]. In some instances, complex introns composed of related introns may allow for various compatible RNA folding arrangements which can facilitate AS (see [138]) enhancing the expression of IEPs. A recent review showed that many different types of “nested” intron arrangements exist in both nuclear, prokaryotic, and organellar genomes, and there might be many mechanisms whereby they can be spliced out [16].

There are reports of twintrons/nested introns from archaeal and eubacterial genomes, organellar genomes in fungi and several plant clades (see Table 1) [16]. Additionally, various euglenoid twintron arrangements have been noted recently, and, interestingly, twintrons in cryptophyte plastids resemble the euglenoid twintrons, suggesting that these elements can move laterally among unicellular eukaryotic algae that gained chloroplasts during secondary endosymbiosis. Zumkeller et al. (2020) reported five instances of twintrons in the mitogenome of lycophytes and hornworts, including an invasive “zombie” hypermobile Group II intron (cox1i1149g2) in Lycopodiaceae that gave rise to two twintrons as an internal intron inserted into itself and into a newly identified succinate dehydrogenase *(sdh3*) intron [257,258]. “Zombie” twintrons are composed of multiple intron modules but splice as one complete unit. A novel twintron configuration was reported in the mitochondrial genome in *Hypomyces aurantius*; in this fungus two Group I introns were arranged side by side (tandem arrangement) within the *cox3* gene [253]. With the rapid accumulation of organellar genome sequences for fungi more mobile introns along with complex intron configurations are bound to be encountered [142,259].

Characterization of the mitogenome for the fungus *Ophiostoma ips* revealed two examples of complex introns: *cytochrome b* (*cob* I4) and the *cytochrome oxidase III* (*cox3* I2) introns. The *cox3* I2 intron is composed of two Group IA1 type intron modules in a possible tandem arrangement, and most likely is excised as a composite intron (‘zombie’ twintron). The complex *cob* I4 intron is the first report of a trintron, which is a complex intron composed of three intron modules. Two Group I introns are in tandem arrangement with the first intron module being interrupted by a Group II intron module. Based on in silico predictions this complex intron could splice by a ratchet-type mechanisms, whereby the upstream Group I intron module splices first followed by the splicing of the downstream Group I intron module. The Group II intron module interrupts the ORF of the first intron module and the Group II module is assumed to splice independently of the other intron modules [142].

Atypical Group II introns and twintrons have been identified in the plastid genome of the freshwater euglenoid *Monomorphina aenigmatica* [260]. Through comprehensive sequencing of the complete plastid genome, it was reported that the ongoing process of intron insertions resulted in intron gain and rapid intron proliferation. Twintrons are assumed to have arisen by a resident intron being invaded by a second intron [261]. To be a successful merger the resulting complex intron has to be processed from the transcript by means of the ribozyme activity of the intron modules and various intron and host genome encoded protein factors.

Twintron/complex introns offer new ribozyme scaffolds that could be engineered where the expression of intron encoded ORFs can be regulated by the splicing of nested ribozyme type components [16]. An example can be found in the complex intron mS1247 from the thermophilic fungus, *Chaetomium thermophilum* var. *thermophilum*, in which the splicing of the internal Group II intron reconstitutes a HE ORF of the external (resident) Group I intron, facilitating its expression which, in turn, contributes to the mobility of the twintron to cognate intron-less *rns* genes [262]. It was also demonstrated that this complex intron could be expressed and spliced in *E. coli*; within *E. coli*, the expressions of the intron encoded protein could be manipulated by modulation the splicing of the internal Group II intron module by manipulating Mg^2+^ concentrations in the media [255]. Essentially the internal intron module can be utilized as an on–off switch for the expression of intron-encoded heterologous proteins in *E. coli*.

The study of complex introns is interesting from an evolutionary point of view, with these configurations evolving independently multiple times involving different categories of introns, to generate complexity. These complex ribozymes my evolve into platform of alternative splicing or as regulatory elements that can modulate the expression of the host gene [211,263]. They may represent intermediate phases that favor the formation of new composite mobile elements and provide an avenue for introns that lack ORFs to achieve mobility by essentially receiving a free ride from the resident intron that encodes a functional HE.

## 11. Conclusions

Exploring organellar genomes and their intron complements is important as these introns can impact genome sizes, genome architecture, and organellar genome diversity. Organellar introns have the potential to serve as regulatory elements that can impact gene expression and, in some instances, introns are associated with a phenotype [207,269]. Mutations impacting splicing efficiencies of organellar introns can lead to respiratory defects and chloroplast defects impacting the physiology and viability of the host cells housing such mutations. The requirement of intron and host genome encoded maturases and splicing factors for most cis- or trans-splicing organellar introns hints at the interconnection between nuclear gene and organellar gene expression.

Polymorphism in mitochondrial Group I introns have been linked to drug susceptibility in fungal pathogens and are being explored as important markers in clinical research [270]. Trans-splicing Group I introns in *Tetrahymena thermophila* can function in RNA repair by replacing mutated regions of mRNA with corrected sequences and, thus, contribute to therapeutic strategies [271]. Group I introns can also function as trans-cleaving ribozymes, that can cause gene inactivation, and help in removing disease associated host mRNA or viral transcripts (reviewed in [270]). Organellar introns and their splicing activity have been viewed as therapeutic targets against fungal pathogens [270,272]. For example, it has been found that targeting Group I and Group II introns with high-affinity small molecules can result in an effective antifungal strategy when dealing with human pathogens [273,274]. In addition, intron RNAs based ribozymes ([268,269,270,271,272,273,274]; reviewed in [275,276]), complex introns (i.e., potentially co-operating ribozymes) and IEPs have applications in biotechnology as RTs, endonucleases, genome editing tools and regulatory switches to control gene expression [25,255,256,277,278].

Group I and II introns, complex introns, and trans-splicing introns could be considered examples of constructive neutral evolution where complex systems evolved by non-adaptive mechanisms (i.e., drift) [279,280]. Possibly originating in the RNA world, organellar ribozyme-based introns are ancient and were maintained in some lineages during the transition of endosymbionts into the formation of organelles such as mitochondria and chloroplast. Group I introns are potential ancestors to the tRNA (BHB) introns and Group II introns are potential ancestors to the nuclear spliceosomal introns. Organellar introns encode proteins that have been coopted as recombinases, RTs, telomerases, spliceosomal RNA, and protein components. Further studies on mitochondrial and chloroplast genomes are warranted as Group I and II introns and their IEPs are building blocks that have contributed to both organellar and nuclear genome evolution and they can impact the expression of genes and they have applications in biotechnology.

## Figures and Tables

**Table 1:** Examples of complex introns composed of self-splicing organellar intron modules across the three kingdoms: plant, algae, and fungi.

Twintron Category	Kingdom	Organism	Host Gene	Subcellular Location	Reference
GI/GI	Fungi	*Cryphonectria parasitica*	*rns*	Mitochondria	[254]
GI/GI	Fungi	*Annulohypoxylon stygium*	*nad5, cob, cox1*	Mitochondria	[259]
GI/GI	Fungi	*Mycogone perniciosa*	*nad1*	Mitochondria	[72]
GI/GI ^1^	Fungi	*Hypomyces aurantius*	*cox1*	Mitochondria	[253]
GI/GI ^1^	Fungi	*Endoconidiophora resinifera*	*rns*	Mitochondria	[141]
GI/GII	Fungi	*Chaetomium thermophilum*	*rns*	Mitochondria	[254]
GI/GII	Fungi	*Grosmannia piceiperda*	*rnl*	Mitochondria	[264]
GI/GII	Fungi	*Chaetomium thermophilum*	ms1247 (position)	Mitochondria	[262]
GI/GII	Fungi	Ascomycota: multiple	*cytb*	Mitochondria	[138]
GI/GII ^2^	Plant	Lycopodiaceae: multiple	*cox1*	Mitochondria	[257]
GI/GII ^3^	Fungi	*Ophiostoma ips*	*cox3, cob*	Mitochondria	[142]
GII/GII	Algae	*Euglena gracilis*	*psbF*	Chloroplast	[13]
GII/GII	Algae	*Euglena gracilis*	*ycf8*	Chloroplast	[265]
GII/GII	Algae	*Pyrenomonas salina*	*cpn60*	Chloroplast	[266]
GII/GII	Algae	*Rhodomonas* sp.	*groEL*	Chloroplast	[267]
GII/GII	Algae	*Porphyridium purpureum*	*atpI, rpoC*	Plastid	[268]
GII/GII	Algae	*Eutreptiella pomquetensis*	*rpoB, psbD, petG*	Plastid	[48]
GIII/GIII	Algae	*Euglena gracilis*	*rps18*	Chloroplast	[15]
GIII/GII	Algae	*Euglena gracilis*	*rps3*	Chloroplast	[13]
GII/GIII, GII/GII, GIII/GIII	Algae	*Euglena gracilis, Monomorphina aenigmatica*	*psb*, *rpoC1*, *rps3*	Plastid	[260]

^1^ tandem Group I introns (side-by-side arrangement), ^2^ novel hypermobile/zombie Group II intron invading itself, ^3^ trintron with three modules (upstream Group I interrupted by Group II, downstream Group I).

## Data Availability

The data and information presented in this review have all been cited to the best of our knowledge.
